# The relationship between parental disability and child outcomes: Evidence from veteran Families

**DOI:** 10.1371/journal.pone.0275468

**Published:** 2022-11-09

**Authors:** Leah K. Lakdawala, Prashant Bharadwaj

**Affiliations:** 1 Department of Economics, Wake Forest University, Winston-Salem, NC, United States of America; 2 Department of Economics, University of California, San Diego, La Jolla, CA, United States of America; University of Notre Dame Keough School of Global Affairs, UNITED STATES

## Abstract

We examine the relationship between parental disability and child outcomes in the American Community Survey. We focus on families with veteran parents, for whom parental disability is a direct result of service-related activities and thus is more plausibly exogenous to child outcomes than other forms of parental disability. Using the service connected disability rating (SCDR) as a measure of the severity of veteran disability, we document a gradient in child outcomes with respect to parental disability (even conditional on having a disabled parent). Children with more severely disabled parents are more likely to be late for grade, less likely to be in private school, and more likely to have disabilities themselves. These results lend meaningful insight to broader populations; we find similar associations between parental disability and child outcomes in non-veteran families. We provide evidence consistent with two broad mechanisms: first, parental disability reduces parental labor supply and thus household income (even net of transfers) and second, children—especially older children—allocate time away from work and schooling to provide care for disabled parents.

## 1 Introduction

The question of how parental disability status affects child outcomes is of critical importance. A rich literature has shown that children’s environments have large impacts on childhood development and can affect health and labor market outcomes in the long run [1, 2]. Yet we know little about the consequences of parental disability for children.

Parental disability can potentially disrupt the accumulation of children’s human capital in a variety of ways. First, disability is strongly correlated with poverty in both developed and developing countries [3–7]. For example, in the U.S., working age adults (ages 18–64) with a disability are more than twice as likely to be in poverty (25% compared to 9.9%; authors’ own calculations using the 2019 American Community Survey (ACS); individuals are considered disabled if they report at least one disability in the following categories: cognitive, physical, vision, hearing, self-care or independent living difficulty; poverty status is defined as having family income below 100% of the poverty line as reported in the ACS). Disability can simultaneously preclude parents from working (a large body of economics research has found that disability negatively affects labor market outcomes; see [4] for a review of this literature) and increase household expenses if disabled parents require increased care. This reduction in financial resources can affect the health care that children in the household receive. This is important for both physical and cognitive disabilities, as early diagnosis and treatment is often critical for limiting the severity of disability [8, 9]. As a result, child health is strongly related to household income [10–13].

Second, parental disability can impact the quality of the home environment. A large body of work documents the effects of children’s environments on their health and educational outcomes (see, for example, [14–16]). In the context of parental disability, one potential pathway works through the disabled parent’s need for additional care. When this care is provided by non-disabled spouse, it reduces the time and resources the spouse can allocate to children [17] as well as the ability to supply labor [18]. When such care is provided by children, qualitative work has found that this has a negative effect on children’s development and childhood experience [19]. Parental cognitive disabilities and injuries such as traumatic brain injury and post-traumatic stress disorder (PTSD) may be particularly harmful to children; for example, PTSD has been associated with higher levels of family violence, marital conflicts, and family distress [20]. Additionally, [21] find that parental disability is associated with lower educational expectations (on the part of both parents and youths). Hence, if parental disability places families in poverty or otherwise disrupts schooling and reduces investments in children, it could have long run consequences that may be difficult or expensive to undo.

To better understand the link between parental disability and child outcomes, this paper examines the empirical relationship between parental disability among veterans and child outcomes at a national level using 12 years of data from the American Community Survey (ACS). Veterans form a considerable proportion of the American population (around 6.8% of the adult population) and have some of the highest rates of disability: according to the 2019 ACS, while around 14.6% of non-veteran adults are disabled, the rate is double (30.7%) among veterans. Given the high rate of disability among veterans, it is unsurprising that children of veterans are especially vulnerable to parental disability; while 8.4% of all children under the age of 18 live with at least one disabled parent in the broader population, that figure jumps to 18.1% when considering children of veterans (authors’ calculations using the 2019 ACS, using the definition of disability above). Thus, in order to shed light on the intergenerational consequences of disability in this population, it is vital to understand how veteran disability affects children’s health and schooling outcomes.

One of the empirical difficulties of examining the impacts of parental disability on child outcomes is that parental disability might be correlated with other attributes that could also affect child outcomes. While exogenous variation in disability status is difficult to find in the general population, we can get closer to the (statistical) ideal of random assignment of disability by examining parental disability in veteran families. Eligible veterans are assigned a “service connected disability rating” (henceforth, SCDR), which ranges from 0–100% and represents the extent of disability due to military service. Since this measure attempts to capture disability specifically due to military service (and not preexisting conditions or disability due to other sources), it is less likely to be driven by underlying unobservable factors otherwise correlated with child outcomes; military disability often results from combat service, which [22] argue is conditionally exogenous for soldiers. For example, disabilities due to service-related injuries are less likely to reflect confounding background characteristics such as parental education, which is likely to be correlated with both parental disability and with children’s schooling investments. Thus, by focusing on the sample of children living with veteran parents, we argue that conditional on having a parent that selected into military service, the degree of the parent’s service-related disability is plausibly exogenous. This is similar to the source of identification used in [23], who find that wartime wounds from World War II service affected veterans’ subsequent labor market outcomes and even the long-run outcomes of their adult children. Moreover, our setting allows us to compare outcomes of children with more and less severely disabled parents, conditional on having a disabled parent. This is important, as it allows us to further limit the bias that results from parents “selecting into” disability.

We find that children (aged 5–18) living with a veteran parent are significantly worse off along schooling and health dimensions when their parent is severely disabled, relative to children whose parent is less severely disabled and to children in families where neither parent is disabled. A child whose parent has the highest disability rating is 6.5% more likely to be late for grade and 48% more likely to report cognitive difficulties compared to a child whose parent has no disability rating. The gradient in child outcomes with respect to parental disability is very steep and outcomes for children with more severely disabled parents are statistically and meaningfully different than for children whose parents are less severely disabled (but are still disabled). These associations point to the idea that children of more severely disabled veterans are likely to enter adulthood at a disadvantage. Moreover, the level of benefits given to a veteran increase as the SCDR increases; since our results do not condition on the receipt of such benefits, these effects can be interpreted as the *overall* effect of SCDR status (including any benefits that veterans receive). Therefore, despite the higher level of benefits that accrue to more disabled veterans, their children appear to be worse off on the dimensions we can measure.

These findings do not appear to be driven by differences in characteristics of families with more or less severely disabled parents, nor do they seem due to differential selection into parenthood or living with children following disability. Interestingly, we find very little heterogeneity in this relationship across children’s race and sex.

We also show that effects of parental disability on children are not limited to the veteran population. In order to get a sense of these relationships in a broader context, we compute correlations between parental disability status and child outcomes in non-veteran families. Within this population, we do not have data on the extent of disability and are hence constrained to only examining outcomes by parental disability status—i.e., whether or not a parent in the household is disabled. Thus we compare correlations between an indicator for parental disability and child outcomes across veteran and non-veteran families. We find that the negative associations between parental disability status and child outcomes are similar for veteran and non-veteran families, though the relationship is stronger in the non-veteran population, where disability is less likely to be exogenous with respect to children’s outcomes.

We also provide evidence that the adverse consequences of parental disability operate in part through two broad channels. First, we show that household income per capita declines sharply with parental disability. This is driven by large reductions in a parent’s labor supply and earnings as that parent’s disability is more severe. Though Veterans Administration (VA) and Supplemental Security Income (SSI) transfers increase with parental disability, they are not enough to offset the total decline in household earned income per capita; thus, on net, children of more severely disabled parents live in poorer households. Second, we illustrate that teens—who are more likely to be capable of providing care for disabled parents compared to younger siblings—are less likely to work when their parents are more severely disabled. Moreover, we find that working teens take jobs that involve shorter commutes when their parents are severely disabled. These findings are both consistent with parental disability requiring care that is often provided by older children in the household. Indeed, the negative relationship between work and schooling outcomes is concentrated among high school-aged children (ages 14–18) and in families where the parental disability explicitly requires care.

To our knowledge, ours is the first paper to quantify the relationship between the degree of parental disability and childhood wellbeing. Several studies compare that children of disabled parents to those of non-disabled parents and find that children of disabled parents tend to fare worse on a range of outcomes. However, these studies focus only on comparing children of disabled versus non-disabled parents (rather than the gradient of child outcomes with respect to the severity of parental disability) and the degree to which they can address the endogeneity of parental disability varies across studies (see [21, 24, 25] for the US and [26] for Vietnam). Recent work has illustrated that parental disability can have long-reaching effects on the adult socioeconomic status and mortality of the next generation [23], and our results provide evidence for pathways through which these effects arise; children of severely disabled parents are at a disadvantage at early ages along the dimensions of schooling and health. In this way, our results are useful for understanding the intergenerational transmission of health shocks more generally. The literature documenting the intergenerational transmission of socioeconomic status and health is vast; for an example, see [27].

Our paper also contributes to the literature on the determinants of childhood disability (see [28] for a summary of some recent literature). Specifically, our results illustrate that parental disability is one important factor that influences child disability, over and above potential genetic transmission of disability. [29] estimate that a family with a disabled child faces disability-associated costs of $30,500 per year and 7.1 million children (14%) in public schools receive special education services costing about $50 billion annually [30, 31]. Given the importance of childhood disability to families and to governments, understanding the link between childhood and parental disability is critical.

Finally, the results in this paper are relevant to the broader research agenda that seeks to understand the effects of parental and family health shocks. A vast body of previous work has established that parental illness and death have critical effects on the wellbeing of the household and household members (see, for example, [32–35]); more recent work also shows that siblings’ disability can impact children’s schooling decisions [36]. Our findings add to this literature by showing that parental disability resulting from military service acts as a significant shock to veteran households and accordingly disrupts the accumulation of children’s human capital.

## 2 Data

The results we present in this paper use data from the American Community Survey (ACS) for the years 2008–2019, the years in which the question of service connected disability rating (SCDR) is asked of veterans. We accessed the data through IPUMS USA [37]. (The underlying data source is the U.S. Census Bureau; the analysis in this paper complies with the IPUMS USA terms and conditions.) The ACS is a 1-in-100 national random sample of the population. One adult from each household responds to survey questions on behalf of all household members, including children. Our main sample is formed of all children of the household head between the ages of 5 and 18 (inclusive) who reside with at least one veteran parent. We do not study children over the age of 18, as the survey only contains information for coresident parents. Thus as age increases, the sample of individuals for which we observe parental disability is likely to become less and less representative of the population. These sample restrictions leave us with over 481,000 children across 12 survey years. For the analysis that uses non-veteran families, our sample includes all children of the household head between the ages of 5 and 18, resulting in over 5.1 million children.

Our main covariate of interest is parental SCDR. SCDR “connotes many factors but basically it means that the facts, shown by evidence, establish that a particular injury or disease resulting in disability was incurred coincident with service in the Armed Forces, or if preexisting such service, was aggravated therein” (38 CFR 3.303). Conditions that determine eligibility typically exclude, “the result of the veteran’s own willful misconduct or, for claims filed after October 31, 1990, the result of his or her abuse of alcohol or drugs” (38 U.S.C. 105). The SCDR is meant to represent a composite measure of both the severity and the connectedness of the disabilities to service. This score is typically calculated when a veteran applies for disability compensation after having undergone a medical exam at a VA hospital. The SCDR reflects both physical disabilities (such as amputations or sensory impairment) and non-physical disabilities (such as post-traumatic stress disorder, PTSD). The SCDR and household demographics determine the level of benefits for which a veteran is eligible; these benefits generally increase linearly with SCDR with the exception of benefits tied to SCDRs of 100%, which are much more generous (see S1 Fig). Though the score is reported to veterans and relevant administrators in increments of 10 percentage points, in the ACS we observe the SCDR only in bins of 20 percentage points and it is top-coded at 70 percent. Veterans with certain severe disabilities or disabilities with “special circumstances such as the need of aid and attendance by another person or by specific disability, such as loss of use of one hand or leg” may be eligible for additional special monthly compensation (SMC) “paid based on the need of aid and attendance by another person” (as reported by the VA, http://www.benefits.va.gov/). Additionally, surviving dependents of veterans who died due to service-related disabilities are eligible to receive Dependency and Indemnity Compensation (DIC). In this analysis we restrict our attention to children in households with living but disabled veterans, so the large majority will not be eligible for DIC.

We also observe some dimensions of general self-reported disability in the ACS for all individuals, including non-veterans. These are indicators for whether an individual has any (i) cognitive difficulties—difficulty learning, remembering, concentrating, or making decisions because of a physical, mental, or emotional condition; (ii) physical difficulties—limitations on “basic” physical activities, such as walking, climbing stairs, reaching, lifting, or carrying; (iii) “long-lasting” condition of blindness, deafness, or a severe vision or hearing impairment; and (iv) self-care and independent living difficulties—inabilities to care for oneself (not including temporary health conditions such broken bones or pregnancy) either within (self-care) or outside (independent living) the home. The ACS does not contain information on the severity or number of disabilities. We observe only the number of disability categories that apply to each individual. It is possible for a person to have multiple disabilities that fall into the same category.

**Fig 1:**
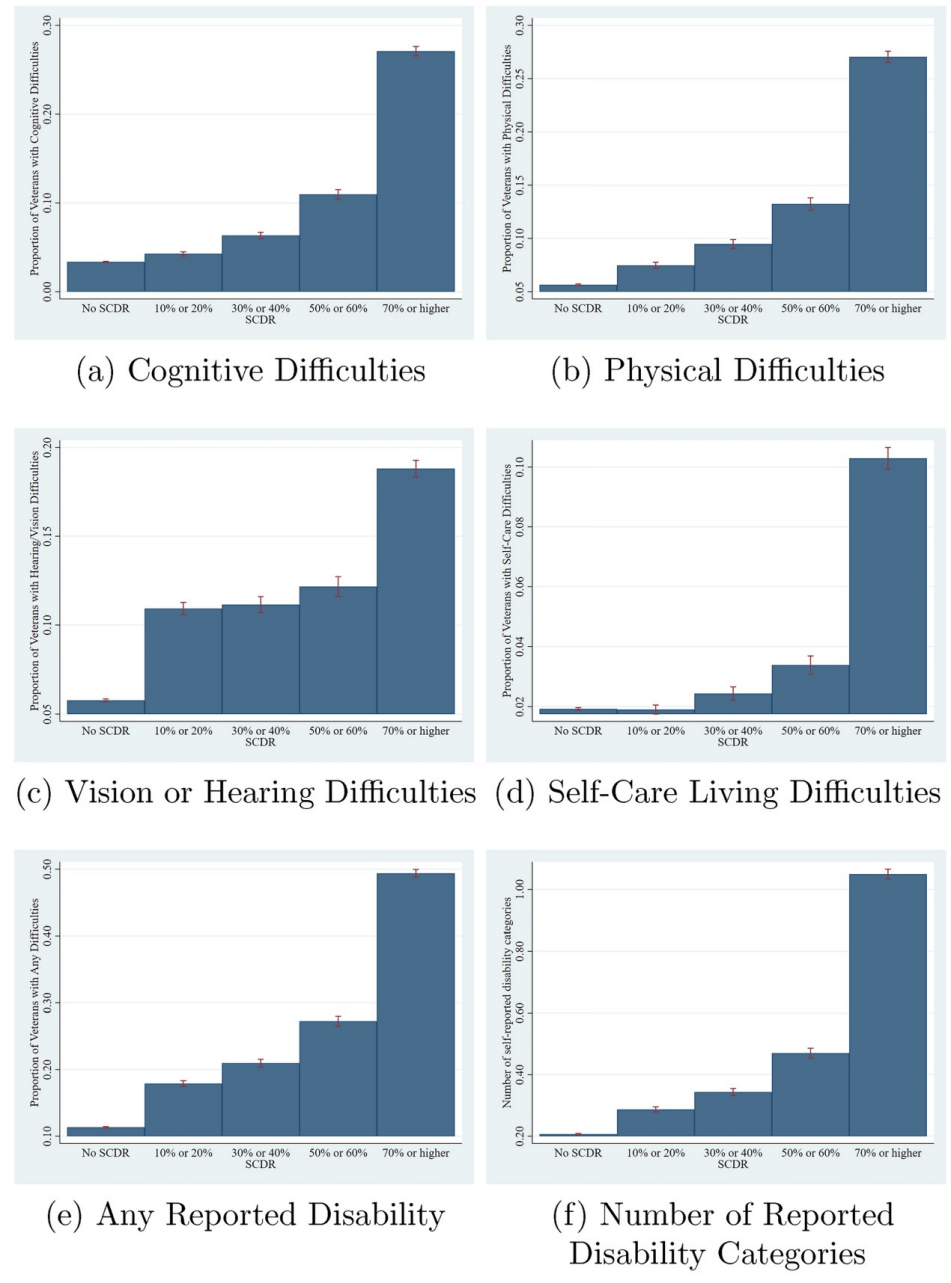
SCDR and self-reported disabilities among veterans. (a) Cognitive Difficulties, (b) Physical Difficulties, (c) Vision or Hearing Difficulties, (d) Self-Care Living Difficulties, (e) Any Reported Disability and (f) Number of Reported Disability Categories. Sample for all figures: all veterans from families included in main estimation sample, i.e. containing at least one child aged 5-17. Number of disability categories in Fig 1f refers to a count of self-reported disability categories, not the total number of disabilities (which is not given in the ACS).

In our sample, self-reported disability and SCDR are highly correlated among veteran parents (Fig 1(a)–1(d)). Self-reported disability across all categories is increasing in SCDR, with a discrete jump up at SCDRs of 70 percent or higher (potentially due to the top-coding of SCDRs in the ACS). The likelihood of reporting any disability and the number of reported disability categories is also increasing in SCDR (Fig 1(e) and 1(f)). On average, those with an SCDR of 70% or higher have disabilities that span more than one category. However, self-reported disability does not perfectly correspond with SCDRs. For example, about 10% of parents without any SCDR report disability (Fig 1(e)); this is because SCDRs apply only to disabilities sustained or worsened due to military service and thus excludes non-service-related disabilities. Additionally, not all individuals with SCDRs self-report disabilities. This could be because the categories of self-reported disability in the ACS do not cover all disability types. Nonetheless, given the strong correlations between self-reported disability measures (including self-reporting disabilities in multiple categories) and SCDR presented in these figures, we believe that variation in parental SCDR captures a combination of the likelihood and severity of parental disability.

Tables 1 and 2 display summary statistics for the samples of children of veteran and non-veteran families. We present statistics for both samples because, as discussed in Section 3, we examine correlates of parental disability for both samples of children. Column (1) shows that about 19% of children have a parent with an SCDR, conditional on living with a veteran parent. In the case where we observe both parents with an SCDR, we use the higher SCDR. About 7% of children live with a veteran with an SCDR of 10–20% (which we refer to as having a less severely disabled parent from this point onwards), and 7% of children live with a parent with the highest SCDR rating (70% and above), which we refer to as having a “severely disabled” parent from this point onwards. Self-reported parental disability is 16% in the veteran family sample (column (1)), which is significantly higher than in the non-veteran sample (9%, column (2)).

**Table 1:** Descriptive statistics for children ages 5–17: Child characteristics.

	Children with at least one veteran parent	Children with non-veteran parents	p-value for *H*_0_: (1) = (2)
	(1)	(2)	(3)
Household Size	4.49	4.50	0.001
	[1.38]	[1.48]	
Number of Siblings in HH	1.45	1.57	0.000
	[1.2]	[1.24]	
Number Grandparents in HH	0.04	0.04	0.000
	[0.21]	[0.23]	
Mother’s Age	41.65	40.40	0.000
	[7.39]	[7.21]	
Mother’s Education			*p- value for the joint test that distribution is the same across groups = 0.000*
High School or Less	0.30	0.38	
1 Year of College	0.17	0.14	
2 Years of College	0.13	0.10	
4 or More Years of College	0.34	0.34	
Missing	0.05	0.04	
Father’s Age	44.99	42.99	0.000
	[8.73]	[7.75]	
Father’s Education			*p- value for the joint test that distribution is the same across groups = 0.000*
High School or Less	0.34	0.35	
1 Year of College	0.20	0.10	
2 Years of College	0.12	0.06	
4 or More Years of College	0.30	0.29	
Missing	0.04	0.19	
Any Parental Disability	0.16	0.09	0.000
Parental SCDR			
No Disability Rating	0.78		
10 to 20 percent	0.07		
30 to 40 percent	0.04		
50 to 60 percent	0.03		
70 percent or more	0.07		
Household Income Per Capita	17742.55	16347.67	0.000
	[15062.6]	[17814.79]	
Number of observations	481,725	5,126,450	

Data from the American Community Survey (2008–2015). Standard deviations in square brackets below means. Household income per capita trimmed at the bottom and top 1% within each survey year and is expressed in 1999 dollars using the CPI-U multiplier published by the Bureau of Labor Statistics. Family income as a percentage of the poverty line is as reported in the ACS, which uses the poverty line established the Social Security Administration in 1964 and subsequently revised in 1980 (adjusted for inflation) as well as detailed income and family structure information. Column 3 reports the p-value for the test that the means across veteran and non-veteran samples are the same. However, due to large sample sizes, the p-values are almost always 0, even when the difference in means is not economically meaningful. Thus, we interpret these p-values with caution.

Children of veterans appear to be very similar to children of non-veterans along most dimensions (e.g. in terms of sex, age, birth order, race, household size, number of siblings, and father’s age). It is worth noting that because of the large sample size, even small differences—which are not economically meaningful—are statistically significant, so we interpret the p-values reported in column 3 with caution. However, there are a few key differences. Children of veterans have slightly older parents and are less likely to be missing information on father’s education than children of non-veterans. This is because children of veterans are much more likely to be living with their fathers (96.2% versus 81.1%). They also have higher household income per capita (by about $1400). Household income per capita is winsorized at the 99.5th percentile within each state and survey year and is expressed in 1999 dollars using the CPI-U multiplier published by the Bureau of Labor Statistics.

The main child outcomes we consider are current schooling, late-for-grade, work, and child disability status across a number of indicators. “Currently in School” is an indicator that is equal to one if a child has attended a school within the last three months. Additionally, for children who are currently attending school, we observe whether the school they attend is public or private. In the ACS, home-schooling is included under the classification of private schooling as is not separately identifiable. As expected, most children are in school (96.5%); about 12.2% are in private school (Table 2). We use private school attendance as a very broad measure of schooling investment/outcomes, for two reasons. First, children who attend private schools (whether due to selection or quality) perform better than students who attend public schools, along standard dimensions of performance such as test scores [38–42] as well as high school graduation and college attendance [43–45]. Second, private schools are typically more expensive than public schools. According to the most recent report from the National Center for Education Statistics, private school tuition is on average $12,420 per year [46]. Moreover, [47] find that the propensity to attend private school increases with both income and ability, suggesting that private school attendance could reflect greater investment in schooling, higher ability (which could be affected by a parents’ disability status), or both. We classify 4.6% of the sample as “Late for Grade”, which we define as being at least 2 years below the modal grade-for-age in the ACS. This measure is meant to capture slower-than-normal progression through school grades and possible grade retention. We calculate this variable for children 8 and older; compulsory school starting ages are state-specific and vary from age 5 to age 8 (as reported in 2008 by the U.S. Department of Education, Institute for Education Sciences, National Center for Education Statistics (http://nces.ed.gov/)).

**Table 2:** Descriptive statistics for children ages 5–17: Household characteristics.

	Children with at least one veteran parent	Children with non-veteran parents	p-value for *H*_0_: (1) = (2)
	(1)	(2)	(3)
Female	0.49	0.49	0.135
Age	11.97	11.52	0.000
	[3.93]	[3.95]	
Birth Order	1.70	1.73	0.000
	[0.9]	[0.93]	
White	0.73	0.62	0.000
Black	0.12	0.11	0.000
Hispanic	0.12	0.20	0.000
*Schooling and Labor Force Outcomes*			
In School (Previous 3 Months)	0.965	0.965	0.014
Attending Private School	0.122	0.126	0.000
Late for Grade	0.046	0.047	0.000
Employed (Previous Year)	0.269	0.249	0.000
Hours Worked (Previous Year)	460.2	452.9	0.002
	[463.15]	[484.07]	
Hourly Earnings (Previous Year)	6.85	7.18	0.000
	[8.57]	[9.23]	
*Disabilities*			
Cognitive Difficulties	0.044	0.038	0.000
Physical Difficulties	0.006	0.006	0.066
Sensory Difficulties	0.012	0.013	0.002
Self-care Difficulty	0.010	0.009	0.013
Independent Living Difficulty	0.025	0.022	0.000
Any Difficulty	0.057	0.051	0.000
Number of observations	481,725	5,126,450	

Data from the American Community Survey (2008–2015). Standard deviations in square brackets below means. Employment (and thus normalized earnings) information only asked of individuals aged 16 or older. Independent living difficulty is only asked of individuals aged 15 or older. Hourly earnings are trimmed at the bottom and top 1% within each survey year and is expressed in 1999 dollars using the CPI-U multiplier published by the Bureau of Labor Statistics. Column 3 reports the p-value for the test that the means across veteran and non-veteran samples are the same. However, due to large sample sizes, the p-values are almost always 0, even when the difference in means is not economically meaningful. Thus, we interpret these p-values with caution.

There are several labor force outcomes we consider, all of which are only reported for those aged 16 and older. The first is an indicator for whether an individual has been employed in the previous year. Around 27% of the sample ages 16–18 report being employed in the past year at an average of 460 hours per year (about 8.8 hours per week). Earnings are on average close to $7 per hour. Hourly earnings are calculated using reported hours worked in a “usual” week and weeks worked in the past year. However, weeks worked in the previous year are only reported in intervals so the midpoint of each interval is used as is standard [48]. Hourly earnings are winsorized at the 99.5th percentile (across all workers) within each state and survey year and are expressed in 1999 dollars using the CPI-U multiplier published by the Bureau of Labor Statistics.

Child disability status is reported in the survey along the same dimensions as for parents. Cognitive difficulties are the most common in our sample (Table 2), affecting 4.4% of children. Physical as well as “long-lasting” sensory (i.e., vision and hearing) difficulties are much less common (0.6%-1.2% of children). About 1% of the sample suffers from self-care difficulty, while about 2.2% of the sample (aged 15 or older) suffers from independent living difficulties.

## 3 Empirical approach and results

### 3.1 The relationship between the degree of parental disability and child outcomes in veteran families

Our baseline estimates are generated by running the following regression on the sample of children age 5–17 with at least one veteran parent:

$$\begin{array}{ccc} Y_{iht} & = & \beta_{1}\cdot 1\left(SCDR_{ht}=\text{10}\;\text{or}\;\text{20}\;\text{percent}\right)\\ & & \;+\beta_{2}\cdot 1\left(SCDR_{ht}=\text{30}\;\text{or}\;\text{40}\;\text{percent}\right)+\beta_{3}\cdot 1\left(SCDR_{ht}=\text{50}\;\text{or}\;\text{60}\;\text{percent}\right)\\ & & \;\;+\beta_{4}\cdot 1\left(SCDR_{ht}=\text{70}\;\text{percent}\;\text{or}\;\text{higher}\right)+\gamma X_{iht}+\delta_{t}+\mu_{s}+\varepsilon_{iht} \end{array}$$ (1)

where *Y*_*iht*_ is an outcome of interest such as schooling or disability status for child *i* in household *h* in survey year *t*; *SCDR*_*ht*_ is the parental veteran disability score in increments of 20 percentage points and top-coded at 70 percent (as noted in Section 2, in cases where both parents report an SCDR we use the higher of the two scores); and *X*_*iht*_ are child- and household-level characteristics: age FE, gender, dummy variables for single race categories (white, black, Hispanic), household size FE, FE for birth order, FE for number of siblings, FE for number of grandparents in household, mother’s and father’s age, education, and marital status FE as well as indicators for whether mothers and father served in 2001 and later (including indicators for missing parental information), FE for metro status, state FE, survey year FE. Importantly, *X*_*iht*_ contains the demographic information used to determine the VA benefit eligibility. *δ*_*t*_ and *μ*_*s*_ represent survey year and state fixed effects that capture aggregate differences in *Y*_*iht*_ by year and across states. *β*_1_, *β*_2_, *β*_3_, and *β*_4_ capture the difference in *Y*_*iht*_ relative to children of veterans without an SCDR, i.e. those who are less likely to be and/or who are less severely disabled. In practice, many of the outcome variables we consider are binary, so in many cases (1) represents a linear probability model. As we sometimes observe multiple children of the same set of parents, we cluster standard errors at the family level.

Though SCDRs are not randomly assigned, we believe that the sample and type of disability under study help move us closer to causal estimates than existing work for several reasons. First, unlike other types of injuries, injuries sustained during military service captured through the SCDR are likely to be unanticipated and unrelated to most preexisting health measures (recall that the score only reflects injuries sustained during service and worsening of preexisting conditions due to service). Therefore, unlike other measures of disability, the SCDR is likely to capture plausibly exogenous variation in parental disability. Second, the SCDR allows us to examine the gradient of outcomes with respect to a measure of the *severity* of parental disability. Specifically, by comparing ${\hat{\beta }}_{2}$, ${\hat{\beta }}_{3}$, and ${\hat{\beta }}_{4}$ to ${\hat{\beta }}_{1}$, we can better understand how the degree of parental disability matters for child outcomes, conditional on having a parent with at least some degree of disability. This helps account for potential parental selection into disability. It is also worth highlighting that by restricting our sample to children in veteran families (i.e., with at least one veteran parent), we circumvent the issue of selection into military service.

#### Results

Table 3 presents the estimates obtained from Eq 1 when we consider schooling outcomes. There is no systematic relationship between whether a child is currently in school (as of the previous 3 months) and parental SCDR; the point estimates are all very close to zero and precisely estimated. This is perhaps unsurprising, as most the overwhelming majority of children are attending school (96.5%). Conditional on being enrolled in school, children of highly disabled veterans (SCDR ≥ 70 percent) are 0.3 percentage points more likely to be late for grade (about 6.5%) relative to children of non-disabled parents (significant at the 5% level). They are also more likely to be late for grade that children with of less severely disabled parents (SCDR = 10–20 percent) and this difference is statistically significant at the 10% level. Children in school are also significantly less likely to be in private school when their parents are more severely disabled and these differences are large relative to the proportion of children in private school for non-disabled parents (column 3). For example, children whose parents have the highest SCDR are 1.6 percentage points less likely to attend private school (12.8%) than those with non-disabled parents, and this difference is significant at the 1% level; the same is true when comparing children with severely disabled parents to children with less severely disabled parents, indicating that even within children of disabled veterans, the severity of parental disability matters. To the extent private school captures current and past investment in education, it appears that children with more disabled parents receive lower schooling investment.

**Table 3:** Degree of parental disability and schooling outcomes for children of veterans.

	In School	Late for Grade	In Private School
	(All Ages 5–18)	(Ages 7–18 & In School)	(Ages 5–18 & In School)
	(1)	(2)	(3)
*Parental SCDR*			
10 to 20 Percent	0.001	-0.000	-0.002
	(0.001)	(0.001)	(0.002)
30 to 40 Percent	-0.001	-0.001	-0.012***
	(0.001)	(0.002)	(0.003)
50 to 60 Percent	0.000	-0.002	-0.018***
	(0.002)	(0.002)	(0.003)
70 Percent or Higher	0.001	0.003**	-0.016***
	(0.001)	(0.002)	(0.002)
Observations	481,725	415,078	465,053
Mean of dependent variable	0.965	0.0461	0.125
p-value for test that SCDR 10–20 = SCDR 70+	0.630	0.0970	0.000

*** p<0.01,

** p<0.05,

* p<0.1

Standard errors clustered at the household level. Omitted group: Children in families where neither parent has a disability rating (SCDR = 0). Sample for column (1): all children ages 5–18 living with a veteran parent; sample is restricted to children age 7–18 and currently in school in column 2 and age 5–18 and currently in school in column 3. Controls: age FE, gender, dummy variables for single race categories (white, black, Hispanic), household size FE, FE for birth order, FE for number of siblings, FE for number of grandparents in household, mother’s and father’s age, education, and marital status FE as well as indicators for whether mothers and father served in 2001 and later (including indicators for missing parental information), FE for metro status, state FE, survey year FE. Mean is reported for children in families where neither parent has an SCDR.

In Table 4, we show that children of more disabled veterans are also more likely to suffer from disabilities themselves, across a variety of disability types. Children of more severely disabled veterans are significantly more likely to have cognitive difficulties, defined as “serious difficulty concentrating, remembering, or making decisions” due to a “physical, mental, or emotional condition” (column 1). This difference grows with the severity of parental disability, as measured by parental SCDR; children of highly disabled veterans (SCDR ≥ 70 percent) are 2 percentage points more likely to have a cognitive disability than those without disabled parents, representing a 48% increase in the prevalence of cognitive difficulties among children. This finding seems especially relevant, as one important way in which parental disability could affect children is through by disrupting the home environment, which has a potentially meaningful impact on children’s mental and emotional development. There is some evidence that parental disability is related to other child disabilities as well; children living with parents with the highest SCDR category are more likely to suffer from disabilities in all categories (columns 1–3). Given this, it is perhaps not surprising that children are less able to care for themselves (column 4) or live independently (column 5; only for those ages 15 and older) when their parents have SCDRs of 70 percent or more. These final two categories of child disability potentially represent the most severe forms of disability reported in the ACS, as they correspond to physical or mental health conditions lasting 6 months or longer that make it difficult for individuals to “take care of their own personal needs, such as bathing, dressing, or getting around inside the home” [37]. In column 6, we use a composite measure of child disability—whether the child reports a disability in any category—and find a steep gradient in parental disability. Therefore along the dimension of child disability, it again appears that children of highly disabled veterans are at a disadvantage.

**Table 4:** Degree of parental disability and disability in children of veterans.

	Cognitive Difficulty	Physical Difficulty	Sensory Difficulty	Self-Care Difficulty	Independent Living Difficulty	Any Difficulty
	(1)	(2)	(3)	(4)	(5)	(6)
*Parental SCDR*						
10 to 20 Percent	0.004***	-0.000	0.002**	0.001	0.001	0.005***
	(0.001)	(0.000)	(0.001)	(0.001)	(0.002)	(0.001)
30 to 40 Percent	0.007***	0.000	0.002*	0.001	-0.001	0.006***
	(0.002)	(0.001)	(0.001)	(0.001)	(0.002)	(0.002)
50 to 60 Percent	0.008***	0.000	0.003**	0.001	0.002	0.010***
	(0.002)	(0.001)	(0.001)	(0.001)	(0.002)	(0.002)
70 Percent or Higher	0.020***	0.002***	0.006***	0.003***	0.009***	0.025***
	(0.002)	(0.001)	(0.001)	(0.001)	(0.002)	(0.002)
Mean of dependent variable	0.0420	0.00597	0.0114	0.00959	0.0243	0.0543
p-value for test that SCDR 10–20 = SCDR 70+	0.000	0.000	0.000	0.002	0.000	0.000
Observations	481,725	481,725	481,725	481,725	155,927	481,725

*** p<0.01,

** p<0.05,

* p<0.1

Standard errors clustered at the household level. Omitted group: Children in families where neither parent has a disability rating (SCDR = 0). Sample for column 1–4 and 6: all children ages 5–18 living with a veteran parent; column 5 includes only children aged 15 or older. Controls: age FE, gender, dummy variables for single race categories (white, black, Hispanic), household size FE, FE for birth order, FE for number of siblings, FE for number of grandparents in household, mother’s and father’s age, education, and marital status FE as well as indicators for whether mothers and father served in 2001 and later (including indicators for missing parental information), FE for metro status, state FE, survey year FE. Mean is reported for children in families where neither parent has an SCDR.

#### Robustness checks & other results

To lend credibility to our assumption that parental SCDR is exogenous in the population of children living with a veteran parent, we show in S1 Table that important household characteristics are largely balanced across SCDR. There are no apparent systematic patterns in household size, number of grandparents in the household, metro status, mother’s education, child gender, or home language with respect to parental SCDR (columns 1–4 and 6–7). Though some coefficients are statistically significant, the point estimates tend to be small and do not yield a clear pattern—notably, they do not indicate that children of severely disabled parents are different along observable dimensions—and they are not generally jointly significant at conventional levels. In column 5, we see that children of more severely disabled parents have slightly younger mothers, but the difference is very small relative to the average age of mothers; put another way, it seems unlikely that having a mother who is 41 years and 8 months old versus 41 years and 9.7 months old could drive the differences that we find.

Another potential concern is that disability could affect selection into parenthood, i.e., parents that choose to have children following a disability could be different than parents who choose to have children in the absence of a disability. To address this concern, we use the (very limited) information we have on the timing of disability and child age. In S2 Table, we restrict the sample to children who were born before their parents’ last tour of duty. Specifically, we include only children who were born before 2001, but whose parents served in the military in 2001 or later. Because we use information on each individual parent’s theater of war, we present the results separately for children with a veteran father (columns 1 and 3) and with a veteran mother (columns 2 and 4). Note that we do not observe specific dates of service for each parent. Instead, we observe only the general “theatre of war.” All veterans that have served since 2001 are grouped in a single “Global War on Terrorism / post-2001” theater. We underscore that these restricted samples are considerably smaller than our main samples (less than 9% of the main sample for fathers and less than 2% for mothers), so this analysis is not well powered to detect the effect sizes in Tables 1 and 2. That said, even for these smaller, restricted samples—where selection into parenthood following disability is very unlikely—we find that children of more severely disabled parents are significantly and substantively less likely to be in private school, with the exception of column 2, where the point estimate is the same as in our main samples but where we lack precision for statistical significance due to the small sample size. For the outcome of child disability (columns 3 and 4), the effect sizes in the restricted samples are statistically significant only for the highest category of parental disability (70 percent or higher). Altogether, we take the results in S2 Table as suggestive evidence that selection into parenthood does not fully explain our main findings.

In order to be able to link children directly to parental disability status, in our main sample we focus only on children of the household head who are currently residing with a veteran parent. To show that our results are robust to using a less restrictive sample, we run our estimation on the sample of all children currently residing with an adult veteran in S3 Table. In this sample, we examine the effect of any coresiding adult’s SCDR on the outcomes of all children in the household (regardless of familial relationship between the veteran and child(ren)). We find very similar patterns with respect to private school status (column 1) and child disability status (column 2). Children living with more severely disabled adults are significantly and considerably more disadvantaged on these margins.

Parental disability could potentially lead to sample selection if disabled veteran parents are more or less likely to live apart from their children than non-disabled veteran parents because we are only able to match children to their parents’ disability status if they live with their parents. To show that this type of sample selection is not driving our results, we examine the relationship between SCDR and living with children (ages 0–18) in the sample of veterans that are likely to be parents. Specifically, in S4 Table we show that, among veterans age 19–50, there is no systematic or statistically significant relationship between own SCDR and the number of coresiding children. Thus, we find no evidence of this type of sample selection.

Finally, we examine the effects of parental disability across child race and gender. Generally, S5 Table illustrates that there are few statistically significant differences in the effects by race (columns 1–2 and 5–6) or sex (columns 3–4 and 7–8). It does seem that the effects of severe parental disability (SCDR of 70 percent or more) are slightly larger for white children (columns 1 and 5) and for boys (columns 4 and 8), though the effects of other SCDR categories seem similar across age and sex.

The biggest differences arise when looking at the relationship between child outcomes and mother’s versus father’s SCDR (S6 Table). For private school status, the magnitudes are much higher for father’s SCDR than for mother’s SCDR (columns 1–2). Conversely, the effects of mother’s SCDR are much stronger for children’s disability status (coliumns 3–4). This pattern is consistent with the interpretation that father’s disability affects child schooling through an income channel, which affects the budgetary aspects of schooling decisions (e.g. private versus public education), while maternal disability affects child outcomes through other channels, such as mother’s time allocation or influence on the home environment. For example, [17] find that maternal disability lowers parents’ school involvement and is associated with a less enriching home environment. We discuss mechanisms more formally in section 4.

### 3.2 The relationship between parental disability status and child outcomes in the wider population

The findings in Section 3.1 indicate that among children in veteran families, more severe parental disability is associated with poorer outcomes in terms of schooling and the incidence of child disabilities. However, it is important to understand whether this relationship is specific to veteran families, who are observably different from the wider population (see Tables 1 and 2). While we do not observe markers for the degree of parental disability in the non-veteran sample, we do observe the existence of (self-reported) parental disability along the dimensions described in Section 2. In this section, we examine the relationship between child outcomes and parental disability status as captured by the an indicator for any parental disability (i.e. any category of disability for either parent), both in the sample of veteran families and the sample of non-veteran families. To assess whether the relationship between parental disability and child outcomes varies across these sub-populations, we also report the p-value for the test that this relationship is the same across the two samples.

It is important to keep in mind that when we look at the gradient of child outcomes with respect to SCDR in the sample of veteran families, we argue that the degree of parental disability in this subsample is likely to be exogenously determined and unlikely to capture other determinants of child outcomes (e.g. disadvantages that pre-date disability). In the wider sample and using the indicator for self-reported parental disability, this is less likely to hold. For example, the intergenerational correlation of disability in the wider population could reflect causal pathways such as time allocation and household income (as will be discussed in Section 4) or simply compositional differences across children with and without disabled parents. Thus we underscore that these estimates reflect associations rather than causal effects of parental disability. Nonetheless, as they are the first estimates of the relationship between schooling outcomes, child disability outcomes, and parental disability using a large, nationally representative dataset (that we are aware of), we still see them as an important step forward in our understanding of the intergenerational effects of parental disability.

The association between schooling outcomes and parental disability in the full sample of children ages 5–18 are displayed in Table 5. On average, children who have a disabled parent are significantly less likely to be in school, more likely to be late for grade, and less likely to be in private school (conditional on being in school). The magnitudes of these associations are meaningful. For example, children of disabled parents are nearly 36–40% more likely to be late for grade (column 2) and 11–12% less likely to attend private school (column 3). This holds true for both veteran and non-veteran families; the point estimates are similar across the two subpopulations, though they are statistically significantly different at the 5% level for late for grade status (but not for private school attendance).

**Table 5:** Parental disability status and schooling outcomes for children.

*PANEL A: Children with a veteran parent*			
	In School	Late for Grade	In Private School
	(Previous 3 Months)	(Conditional)	(Conditional)
	(1)	(2)	(3)
Parent declares a disability	-0.006***	0.015***	-0.015***
	(0.001)	(0.001)	(0.002)
Observations	481,725	415,078	465,053
Mean of dependent variable	0.967	0.0421	0.127
*PANEL B: Children with non-veteran parents*			
	In School	Late for Grade	In Private School
	(Previous 3 Months)	(Conditional)	(Conditional)
	(1)	(2)	(3)
Parent declares a disability	-0.006***	0.018***	-0.014***
	(0.000)	(0.000)	(0.001)
Observations	5,126,450	4,303,343	4,945,546
Mean of dependent variable	0.966	0.0447	0.129
p-value for *H*_0_: Panel A = Panel B	0.898	0.0208	0.324

*** p<0.01,

** p<0.05,

* p<0.1

Standard errors clustered at the household level. Sample for column (1): all children ages 5–18; sample is restricted to children age 7–18 and currently in school in column 2 and age 5–18 and currently in school in column 3. Controls: age FE, gender, dummy variables for general race categories (white, black, other—omitted; Hispanic—nonexclusive), household size FE, FE for birth order, FE for number of siblings, FE for number of grandparents in household, mother’s and father’s age and education FE (including indicators for missing parental information), FE for metro status, state FE, survey year FE.

Child and adult disability are also strongly correlated in the wider sample (Table 6). Children are much more likely to have disabilities of all types when they have at least one parent with a disability. The coefficients are large and meaningful. Having a disabled parent increases the chances of a child disability by 1.1 to 3.7 times (i.e., 110% to 370% over the incidence of child disability in the population of children without disabled parents). Interestingly, the correlation is notably stronger in non-veteran families than in veteran families (differences are statistically significant across all disability types), despite the fact that rates of child disability among the sample of children with non-disabled parents are very similar across the two groups. This is suggestive evidence that parental disability in veteran parents is more plausibly exogenous and unrelated to underlying differences between disabled and non-disabled parents and lends credibility to the estimates discussed in Section 3.1.

**Table 6:** Parental disability status and disability in children.

*PANEL A: Children with a veteran parent*						
	Cognitive Difficulty	Physical Difficulty	Sensory Difficulty	Self-Care Difficulty	Independent Living Difficulty	Any Difficulty
	(1)	(2)	(3)	(4)	(5)	(6)
Parent declares a disability	0.061***	0.008***	0.021***	0.010***	0.026***	0.079***
	(0.001)	(0.000)	(0.001)	(0.001)	(0.001)	(0.001)
Observations	481,725	481,725	481,725	481,725	155,927	481,725
Mean of dependent variable	0.033	0.005	0.009	0.008	0.020	0.043
*PANEL B: Children with non-veteran parents*						
	Cognitive Difficulty	Physical Difficulty	Sensory Difficulty	Self-Care Difficulty	Independent Living Difficulty	Any Difficulty
	(1)	(2)	(3)	(4)	(5)	(6)
Parent declares a disability	0.080***	0.010***	0.032***	0.012***	0.035***	0.106***
	(0.001)	(0.000)	(0.000)	(0.000)	(0.001)	(0.001)
Observations	5,126,450	5,126,450	5,126,450	5,126,450	1,448,513	5,126,450
Mean of dependent variable	0.030	0.005	0.009	0.008	0.018	0.040
p-value for *H*_0_: Panel A = Panel B 1	0.000	0.000	0.000	0.000	0.000	0.000

*** p<0.01,

** p<0.05,

* p<0.1

Standard errors clustered at the household level. Sample for column (1): all children ages 5–18; column (6) includes only individuals age 15–18. Controls: age FE, gender, dummy variables for general race categories (white, black, other—omitted; Hispanic—nonexclusive), household size FE, FE for birth order, FE for number of siblings, FE for number of grandparents in household, mother’s and father’s age and education FE (including indicators for missing parental information), FE for metro status, state FE, survey year FE.

That the correlations between parental disability status and child outcomes are similar across the veteran and non-veteran populations speaks to the external validity of our findings—namely, that the effects of parental disability are not likely limited to veteran families. However, we recognize that veteran disability may differ from civilian disability along key dimensions. For example, a veteran’s loss of vision due to military service may be very different from the lack of vision in a civilian; in particular, the trauma associated with a loss of vision in wartime settings may yield additional emotional and psychological consequences for veterans. This could mean that the effects of veteran disability on children might be different than the effects of civilian disability. Thus we regard the external validity of our results beyond the veteran population with caution.

## 4 Mechanisms

In this section, we present evidence for two particular channels through which parental disability affects child outcomes: through a reduction in household economic resources and through an increase in the need to care for parents with disabilities.

### 4.1 Income

A long line of research documents the negative effects that disability has on adult labor market outcomes (see [4] for a review). Relatedly, prior work has found that parental job loss can adversely impact children (see, for example, [12] and [13]). Thus, one way in which parental disability could affect child schooling and health outcomes is through its impact on parents’ ability to earn income and on household resources more generally.

To investigate this channel, we first explore the relationship between parental SCDR and household income per capita in Table 7. Children with more severely disabled parents live in households with significantly lower income; household income per capita is $1,185 (6.7%) lower for children with severely disabled parents on net (column 1). This measure of income per capita includes transfers from the Veterans Administration (VA) and other assistance programs, so it suggests that overall economic resources are lower for these children. In fact, when we examine other specific sources of income, we find that income from “other sources”—which explicitly includes payments from the VA, we see that income per capita from this source increases steeply with parental disability, as expected (column 2). Similarly, Supplemental Security Income (SSI) payments also increase with parental disability, though in much smaller amounts (column 3); this reflects the fact that transfers from the VA lower eligibility for other types of assistance, including SSI. Welfare receipt does not seem to be systematically related to parental SCDR. Column 5 illustrates that earned income (income from wages, salary, and owned business and farms) is the driving force behind the lower observed household incomes for children of severely disabled parents. The more severely disabled the parent is, the less earned income in the household, and transfers from the VA and other sources are not enough to fully offset this lost income.

**Table 7:** Degree of parental disability and household income (veteran sample).

	Household Income per capita	Other Income (includes VA payments)	SSI Income per capita	Welfare Income per capita	Earned Income per capita
	(1)	(2)	(3)	(4)	(5)
*Parental SCDR*					
10 to 20 Percent	-248.5***	374.5***	4.15	0.529	-907.0***
	(90.7)	(11.5)	(3.42)	(1.566)	(86.9)
30 to 40 Percent	-433.1***	1,085.0***	8.59**	0.138	-1,841.0***
	(112.1)	(17.3)	(4.35)	(2.155)	(106.6)
50 to 60 Percent	-400.1***	2,054.5***	17.43***	-2.680	-2,978.5***
	(121.5)	(25.3)	(5.32)	(1.910)	(115.6)
70 Percent or Higher	-1,185.1***	4,458.8***	121.76***	0.328	-6,151.4***
	(85.9)	(34.7)	(6.78)	(2.021)	(82.8)
Observations	481,620	481,725	481,725	481,725	481,725
Mean of dep. var.	17796	349.1	72.81	21.02	16514
p-value for test that SCDR 10–20 = SCDR 70+	0.000	0.000	0.000	0.934	0.000

*** p<0.01,

** p<0.05,

* p<0.1

Standard errors clustered at the household level. Omitted group: Children in families where neither parent has a disability rating (SCDR = 0). Sample: all children ages 5–18 living with a veteran parent. Controls: age FE, gender, dummy variables for single race categories (white, black, Hispanic), household size FE, FE for birth order, FE for number of siblings, FE for number of grandparents in household, mother’s and father’s age, education, and marital status FE as well as indicators for whether mothers and father served in 2001 and later (including indicators for missing parental information), FE for metro status, state FE, survey year FE. Mean is reported for children in families where neither parent has an SCDR. All values have been winsorized at the 99.5th percentile across the entire sample (including non-veteran households).

Note that [49] find that earnings losses for veterans with SCDRs are smaller than VA payments on average. However, we believe that our findings—specifically, that household earnings per capita are not fully offset by VA transfers—are consistent with [49] for two main reasons. First, disabled veterans often require care, which reduces the labor supply and earnings of other household members in addition to the disabled veteran herself/himself (we discuss this in more detail in Section 4.2). Thus, total household earnings per capita may fall by more than VA payments even if a veteran’s own earnings losses are less than VA payments. Second, we study the sample of disabled veterans living with children. VA payments take into account household demographics but are most generous on a per capita basis for veterans without dependents (see S1 Fig). Thus even if veterans’ earnings losses are less than VA payments on average, this may not be true on a per capita basis for veterans with families.

It is worth highlighting that our results in Table 7 show that on average, within the group of veteran parents with an SCDR of 70 percent or higher, families experience a large decline in per capita income and a large increase in “Other Income,” which includes VA payments. However, this average effect could mask substantial heterogeneity within this group, particularly for veterans with an SCDR of 100%, who receive considerably higher VA payments (see S1 Fig). For veterans with an SCDR of 100%, we might expect that the increase in “Other Income” is larger and thus the reduction in total household per capita income lower.

In Table 8, we further document the effect of parental disability on parental labor supply. Specifically, we show that, among children with a veteran father, fathers’ probability of work and work hours decline sharply when fathers are more severely disabled (columns 1 and 2). The pattern is strikingly similar for children with a veteran mother (columns 3 and 4) despite the smaller sample sizes. (We display the effects of parental disability on the non-disabled parents’ labor supply in S7 Table and discuss the results in Section 4.2.) The reductions in labor supply are large and meaningful; severely disabled fathers are 32.8 percentage points less likely to work relative to non-disabled veteran fathers (who work at high rates, 92.2%). The gradient is very steep; veteran fathers with a less severe disability (10–20 percent) are only 1.4 percentage points less likely to work than non-disabled fathers and the difference in the effects of severe disability (70 percent or more) are significantly different from the effects of less severe disability (10–20 percent).

**Table 8:** Degree of parental disability and parental labor supply (veteran sample).

	Father Works	Father’s Work Hours	Mother Works	Mother’s Work Hours
	(1)	(2)	(3)	(4)
*Parental SCDR*				
10 to 20 Percent	-0.014***	-48.2***	-0.023***	-58.6***
	(0.002)	(6.9)	(0.008)	(18.6)
30 to 40 Percent	-0.034***	-134.8***	-0.049***	-144.3***
	(0.003)	(9.2)	(0.010)	(22.1)
50 to 60 Percent	-0.078***	-276.7***	-0.097***	-267.7***
	(0.004)	(11.8)	(0.012)	(26.0)
70 Percent or Higher	-0.328***	-859.3***	-0.318***	-721.5***
	(0.004)	(10.0)	(0.011)	(21.5)
Observations	433,903	433,903	73,283	73,283
Mean of dep. var.	0.923	2030	0.775	1384
p-value for test that SCDR 10–20 = SCDR 70+	0.000	0.000	0.000	0.000

*** p<0.01,

** p<0.05,

* p<0.1

Standard errors clustered at the household level. Omitted group: Children in families where neither parent has a disability rating (SCDR = 0). Sample: all children ages 5–18 living with a veteran father (columns 1 and 2) or mother (columns 3 and 4). Controls: age FE, gender, dummy variables for single race categories (white, black, Hispanic), household size FE, FE for birth order, FE for number of siblings, FE for number of grandparents in household, mother’s and father’s age, education, and marital status FE as well as indicators for whether mothers and father served in 2001 and later (including indicators for missing parental information),FE for metro status, state FE, survey year FE. Mean is reported for children in families where neither parent has an SCDR.

### 4.2 Caring for disabled parents

Another way in which parental disability can affect children is through the additional caregiving needs a disabled parent may require. If children devote time to caring for a disabled parent, this may decrease the time they spend on schooling activities (such as homework) and other activities (such as work for older children). This suggests that some of the adverse effects on children documented in Section 3.1 may be due to a reallocation of children’s time toward parental care and away from human capital accumulation. This channel is more likely to be relevant for older children, who are more likely to be capable of providing care.

To explore this mechanism, we first study work outcomes for teens (ages 16 and older, for whom the ACS contains work information). Other than schooling, work outcomes are the only other type of information that the ACS collects with regard to time allocation. In Table 9, we show that teens are 4.6 percentage points less likely to work than teens with a non-disabled parent (column 1). This is a large effect—around 11% over the average work probability of teens without a disabled parent—and it is statistically significant at the 1% level, as is the difference relative to teens with a less disabled parent. Hours of work are also lower for teens with severely disabled parents (column 2) though this appears to be driven by extensive margin changes in work status, as there are no effects on hours conditional on working (column 3). Interestingly, we find that working teens with more severely disabled parents have jobs that require lower transit time (column 4). This is also consistent with the notion that teens that must care for disabled parents have less time to for other activities, including commuting to jobs. Finally, in column 5 we do not observe that parental disability is systematically related to hourly earnings. We regard the results on work hours conditional on working (columns 3–5) as suggestive, as we find that parental SCDR affects work status and we lack a separate instrument for selection into work.

**Table 9:** Degree of parental disability and teen labor supply (veteran sample).

	Works	Work Hours (All)	Work Hours (Workers)	Travel Time (minutes)	Hourly Earnings
	(1)	(2)	(3)	(4)	(5)
*Parental SCDR*					
10 to 20 Percent	0.003	-3.14	-13.0*	-0.230	0.165
	(0.005)	(3.99)	(7.83)	(0.260)	(0.164)
30 to 40 Percent	-0.008	4.22	17.2	0.459	-0.065
	(0.007)	(5.30)	(10.6)	(0.377)	(0.220)
50 to 60 Percent	-0.018**	-4.60	8.23	-0.672*	-0.503***
	(0.008)	(6.33)	(13.2)	(0.402)	(0.180)
70 Percent or Higher	-0.046***	-21.0***	-7.83	-0.836***	-0.277
	(0.006)	(4.37)	(9.85)	(0.320)	(0.175)
Observations	116,001	116,001	46,655	46,655	46,652
Mean of dep. var.	0.409	188.1	460.1	10.77	6.873
p-value for test that SCDR 10–20 = SCDR 70+	0.000	0.001	0.662	0.122	0.049

*** p<0.01,

** p<0.05,

* p<0.1

Standard errors clustered at the household level. Omitted group: Children in families where neither parent has a disability rating (SCDR = 0). Sample: all teenagers ages 16–18 living with a veteran parent; sample is further restricted to working children in columns 3–5. Controls: age FE, gender, dummy variables for single race categories (white, black, Hispanic), household size FE, FE for birth order, FE for number of siblings, FE for number of grandparents in household, mother’s and father’s age, education, and marital status FE as well as indicators for whether mothers and father served in 2001 and later (including indicators for missing parental information), FE for metro status, state FE, survey year FE. Mean is reported for children in families where neither parent has an SCDR. Earnings are winsorized at the 99.5th percentile.

We find corroborating evidence of the time cost of having a disabled family member in S7 Table, where we show that mothers’ labor supply is adversely affected when fathers are more severely disabled (and vice versa for disabled mothers). Mothers are 7.9 percentage points (10%) less likely to work when fathers are severely disabled, and this difference is significant at the 1% level relative to families with non-disabled fathers and families with less severely disabled fathers (column 1). Mother’s hours of work are also lower in families with a more severely disabled father (column 2). The effects are very similar in families with a veteran mother (columns 3–4). Overall, the evidence in Table 9 and S7 Table suggest that more severely disabled parents require additional care, and the time devoted to this care comes at the cost of both teen and spousal labor supply.

We further investigate the role of care for a disabled parent in Table 10. We begin by reproducing the baseline relationship between parental disability status and child schooling status in column 1. We see that children whose parent has a disability is 0.6 percentage points less likely to currently be in school. In column 2, we decompose this average effect by type of parental disability. 6.6% of children in the sample live with a parent with a disability that does not require care while 9.4% of children in the sample live with a parent with a disability that requires care. The negative relationship between parental disability and schooling is strongest for types of parental disability that explicitly require care—disabilities that limit an individual’s ability to perform basic physical, self-care, or mobility activities (i.e., physical, mobility, or self-care disabilities). The coefficient is substantially and significantly higher for parental disabilities that require care (p-value for difference = 0.002). In column 3 we decompose the average effect by child age. Here we see that the negative relationship is entirely driven by high school-age children (ages 14–18), who are most likely to be able to provide care for disabled parents; high school-age children are 1.4 percentage points less likely to currently be in school if they have a disabled parent. In fact, there is no significant relationship between schooling status and parental disability for elementary school-age children (ages 5–10) or middle school-age children (ages 11–13). In column 4, we show that on average, teens (16–18) are 3.2 percentage points less likely to work when they have a disabled parent. In column 5, we show that this relationship is specific to parents who have a disability that requires care; there is no relationship between parental disability and teen labor supply when the disabled parent does not require care.

**Table 10:** Effects of parental disability by type of parental disability and child age (veteran sample).

	In School	In School	In School	Works	Works
	(1)	(2)	(3)	(4)	(5)
Parent has any disability	-0.006***			-0.032***	
	(0.001)			(0.004)	
Parent has a disability that requires care		-0.008***			-0.050***
		(0.001)			(0.004)
Parent has a disability that does not require care		-0.003***			-0.003
		(0.001)			(0.006)
Parent has any disability × Age 5–10			-0.000		
			(0.001)		
Parent has any disability × Age 11–13			0.000		
			(0.001)		
Parent has any disability × Age 14–18			-0.014***		
			(0.001)		
Observations	481,725	481,725	481,725	116,001	116,001
Mean of dep. var.	0.967	0.967	0.967	0.411	0.411
p-value for test that requires care = does not require care		0.002	0.000^1^		0.000

*** p<0.01,

** p<0.05,

* p<0.1

Standard errors clustered at the household level. Omitted group: Children in families where neither parent reports a disability. Disabilities that require care are physical, mobility, or self-care disabilities. Sample: all children ages 5–18 living with a veteran parent; sample is further restricted to teenagers ages 16–17 in columns 4–5. Controls: age FE, gender, dummy variables for single race categories (white, black, Hispanic), household size FE, FE for birth order, FE for number of siblings, FE for number of grandparents in household, mother’s and father’s age, education, and marital status FE as well as indicators for whether mothers and father served in 2001 and later (including indicators for missing parental information), FE for metro status, state FE, survey year FE. Mean is reported for children in families where neither parent has an SCDR.

^1^ p-value for the test that effects of parental disability for children age 5–10 is the same as for children 14–18.

Taken all together, these findings are consistent with the possibility that older children spend more time caring for disabled parents and are thus unable to attend school or work at the same rates as their counterparts with non-disabled or less-disabled parents. This interpretation of the findings aligns with the limited existing evidence on the effect of parental illness on time spent in household chores and caregiving [33, 50, 51]. For example, [33] use data from China and find a strong negative association between parental chronic health conditions and disability and children’s school enrollment, attendance, and performance as well as on educational spending; they find a positive (though not always statistically significant) association between maternal ill health and time spent working in the household.

### 4.3 Locational preferences and access to health care and schooling facilities

Another possible channel through which parental disability affects human capital investments in children is through its impact on the locational preferences of veterans. For example, more severely disabled veterans may not be able to afford to live in neighborhoods with easy access to private schools, and therefore their children may be less likely to attend private schools. Alternatively, more severely disabled veterans may need to locate closer to health care facilities, and thus their children may also have easier access to health care and thus experience improved health outcomes.

To explore this possibility, we show that our results are robust to including county of residence fixed effects in S8 Table. We argue that within counties, access to schooling and healthcare access is more similar across households. Even once we focus on within-county comparisons, we find that more children of more disabled parents are less likely to attend private school and are more likely to have a disability themselves.

## 5 Conclusion

We find evidence that children face disadvantages in terms of schooling and own disability outcomes when a parent is disabled. Importantly, we document that there exists a gradient in child outcomes in the population of veteran families, for whom the degree of parental disability is more plausibly exogenous. This appears to operate at least in part through two channels. First, parental disability lowers household income and thus the resources available to invest in children’s human capital. Second, parents with disabilities can require care, which is likely to be provided by older children in the household, reducing time these teens spend on schooling and work.

We believe this is an important step towards a deeper understanding of the relationship between parental disability and child outcomes, especially for vulnerable populations. Despite the fact that more severely disabled veterans received greater disability benefits, this paper shows that their children are still worse off, implying that disability related social safety nets are perhaps not able to fully insure children in military families. We highlight that these relationships are also likely to hold in the broader population of non-veterans. However, our analysis is limited by data availability; we are only able to study the effects of parental disability on a small set of outcomes that are coarsely measured. Analyzing the impact of parental disability on different facets of child development represents an important avenue for future research.

## Supporting information

S1 Fig Veterans compensation benefits by service connected disability rate and demographics. (PDF)

S1 Table Balance on observable characteristics by SCDR (veteran sample). (PDF)

S2 Table Impacts of parental disability for children born before parent’s disability (veteran sample). (PDF)

S3 Table Effects of any adult SCDR (veteran sample). (PDF)

S4 Table Relationship between SCDR and number of children in the household (veteran sample). (PDF)

S5 Table Heterogeneity by race and sex of child (veteran sample). (PDF)

S6 Table Heterogeneity by the identity of disabled parent (veteran sample). (PDF)

S7 Table Parental disability and the labor supply of the non-disabled parent (veterans sample). (PDF)

S8 Table Effects of parent SCDR controlling for county fixed effects (veteran sample). (PDF)
