# Supplementary material for: The relationship between parental disability and child outcomes: Evidence from veteran Families
Source: PLoS One. 2022 Nov 9;17(11):e0275468. doi: 10.1371/journal.pone.0275468 (PMC9645595; doi:10.1371/journal.pone.0275468)
Supplement: S1 Fig — (PDF) [file pone.0275468.s001.pdf]

Appendix

705

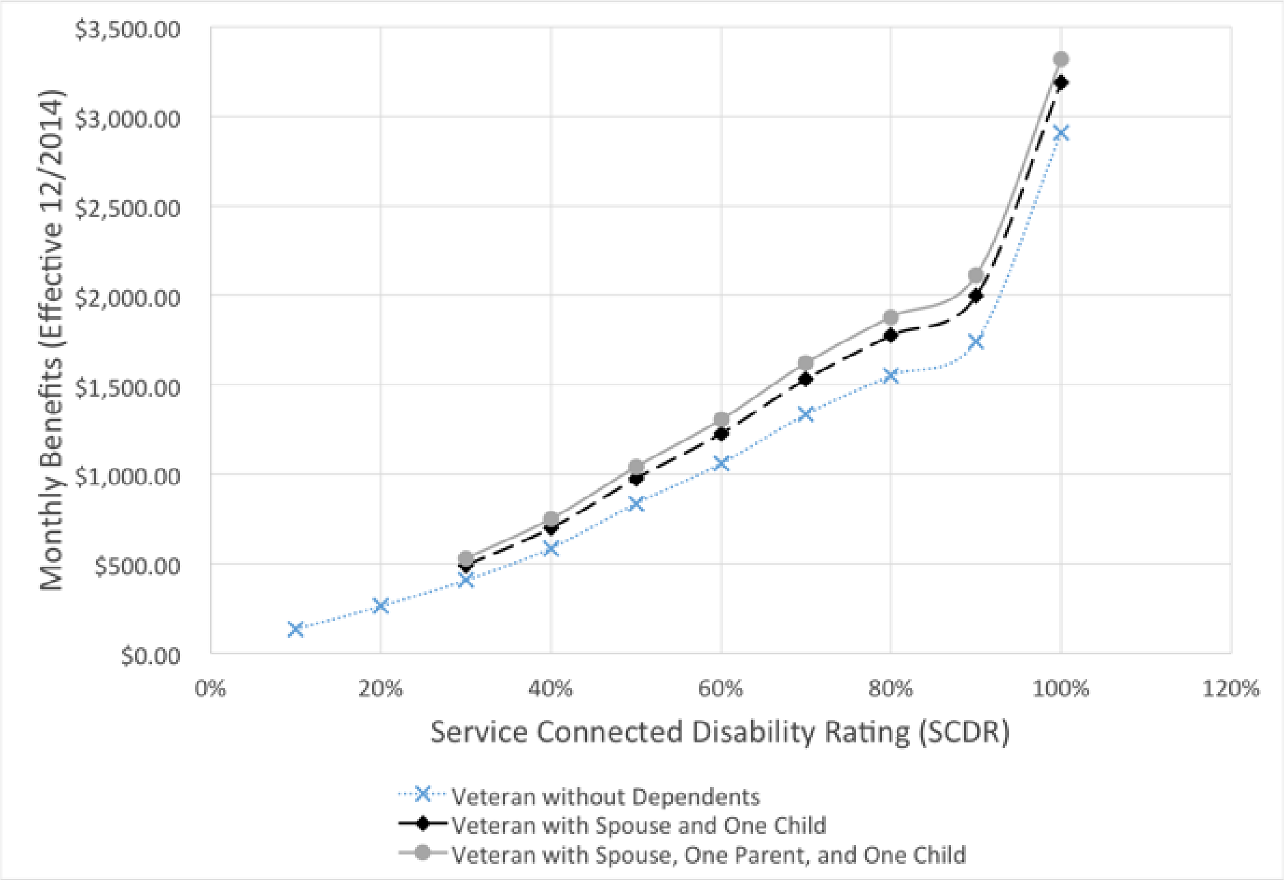

As reported on http://www.benefits.va.gov. Effect as of 12/1/14.

S1 Fig. Veterans Compensation Benefits by Service Connected Disability Rate and Demographics.
