# Supplementary material for: The relationship between parental disability and child outcomes: Evidence from veteran Families
Source: PLoS One. 2022 Nov 9;17(11):e0275468. doi: 10.1371/journal.pone.0275468 (PMC9645595; doi:10.1371/journal.pone.0275468)
Supplement: S1 Table — (PDF) [file pone.0275468.s002.pdf]

| | Household Size (1) | No. of Grandparents (2) | Metro Area (3) | Mother has Coll. Deg. (4) | Mother's Age (5) | Child is Female (6) | English at Home (7) |
|---|---|---|---|---|---|---|---|
| *Parent SCDR* | | | | | | | |
| 10 to 20 Percent | -0.002 | 0.001 | -0.009** | 0.004 | 0.046 | 0.002 | 0.003 |
| | (0.003) | (0.001) | (0.004) | (0.004) | (0.034) | (0.003) | (0.002) |
| 30 to 40 Percent | -0.008* | 0.002 | -0.008 | 0.008* | -0.094** | 0.001 | 0.003 |
| | (0.004) | (0.002) | (0.005) | (0.004) | (0.044) | (0.004) | (0.002) |
| 50 to 60 Percent | 0.011** | -0.003 | -0.008 | -0.001 | -0.088* | -0.005 | 0.002 |
| | (0.005) | (0.002) | (0.006) | (0.005) | (0.052) | (0.004) | (0.003) |
| 70 Percent or Higher | 0.004 | 0.002 | -0.004 | -0.004 | -0.140*** | 0.002 | -0.003 |
| | (0.004) | (0.002) | (0.005) | (0.004) | (0.042) | (0.003) | (0.002) |
| Observations | 481,725 | 481,725 | 411,386 | 455,946 | 455,946 | 481,725 | 481,725 |
| Mean of dep. var. | 4.496 | 0.0344 | 0.459 | 0.675 | 41.81 | 0.485 | 0.931 |
| p-value for joint test of SCDR indicators | 0.0410 | 0.219 | 0.101 | 0.216 | 0.001 | 0.607 | 0.121 |

*** p<0.01, ** p<0.05, * p<0.1 Standard errors clustered at the household level. Omitted group: Children in families where neither parent has a disability rating (SCDR=0). Sample: all children ages 5-18 living with a veteran parent. Controls (included in all columns except when used as an outcome): age FE, gender, dummy variables for single race categories (white, black, Hispanic), household size FE, FE for birth order, FE for number of siblings, FE for number of grandparents in household, mother's and father's age, education, and marital status FE as well as indicators for whether mothers and father served in 2001 and later (including indicators for missing parental information), FE for metro status, state FE, survey year FE. Mean is reported for children in families where neither parent has an SCDR.

**S1 Table. Balance on Observable Characteristics by SCDR (Veteran Sample).**
