# Supplementary material for: The relationship between parental disability and child outcomes: Evidence from veteran Families
Source: PLoS One. 2022 Nov 9;17(11):e0275468. doi: 10.1371/journal.pone.0275468 (PMC9645595; doi:10.1371/journal.pone.0275468)
Supplement: S4 Table — (PDF) [file pone.0275468.s005.pdf]

	Number of children in the household (1)
Own SCDR	
10 to 20 Percent	0.003
	(0.006)
30 to 40 Percent	-0.006
	(0.007)
50 to 60 Percent	0.006
	(0.009)
70 Percent or Higher	0.006
	(0.007)
Observations	571,668
Mean of dep. var.	1.016
p-value for test that SCDR 10-20 = SCDR 70+	0.741

*** p<0.01, ** p<0.05, * p<0.1 Robust standard errors reported. Omitted group: veterans without a disability rating (SCDR=0). Sample: all veterans ages 19-50. Controls: age FE, gender, dummy variables for single race categories (white, black, Hispanic), and education as well as FE for metro status, state FE, survey year FE. Mean is reported for veterans without a disability.

S4 Table. Relationship between SCDR and number of children in the household (Veteran Sample).
