# Supplementary material for: The relationship between parental disability and child outcomes: Evidence from veteran Families
Source: PLoS One. 2022 Nov 9;17(11):e0275468. doi: 10.1371/journal.pone.0275468 (PMC9645595; doi:10.1371/journal.pone.0275468)
Supplement: S5 Table — (PDF) [file pone.0275468.s006.pdf]

	Dependent Variable: In Private School				Dependent Variable: Any Disability			
	White	Non-white	Female	Male	White	Non-white	Female	Male
	(1)	(2)	(3)	(4)	(5)	(6)	(7)	(8)
Parent SCDR								
10 to 20 Percent	-0.002	-0.002	-0.001	-0.004	0.004**	0.005**	0.004**	0.005***
	(0.003)	(0.004)	(0.003)	(0.003)	(0.002)	(0.003)	(0.002)	(0.002)
30 to 40 Percent	-0.012***	-0.011**	-0.009**	-0.015***	0.007***	0.004	0.005**	0.008***
	(0.004)	(0.005)	(0.004)	(0.004)	(0.002)	(0.003)	(0.002)	(0.003)
50 to 60 Percent	-0.017***	-0.019***	-0.019***	-0.018***	0.009***	0.010**	0.009***	0.011***
	(0.004)	(0.005)	(0.004)	(0.004)	(0.003)	(0.004)	(0.003)	(0.003)
70 Percent or Higher	-0.020***	-0.008**	-0.013***	-0.019***	0.028***	0.020***	0.021***	0.030***
	(0.003)	(0.004)	(0.003)	(0.003)	(0.002)	(0.003)	(0.002)	(0.003)
Observations	337,862	127,191	226,353	238,700	349,921	131,804	233,934	247,791
Mean of dep. var.	0.134	0.100	0.127	0.123	0.0543	0.0543	0.0413	0.0665
p-value for test that								
SCDR 10-20 = SCDR 70+	0.000	0.261	0.004	0.000	0.000	0.000	0.000	0.000

*** p<0.01, ** p<0.05, * p<0.1 Standard errors clustered at the household level. Omitted group: Children in families where neither parent has a disability rating (SCDR=0). Sample: all children ages 5-18 living with a veteran parent; further sample restrictions listed above each column. Controls (included in all columns except when used as an outcome): age FE, gender, dummy variables for single race categories (white, black, Hispanic), household size FE, FE for birth order, FE for number of siblings, FE for number of grandparents in household, mother's and father's age, education, and marital status FE as well as indicators for whether mothers and father served in 2001 and later (including indicators for missing parental information), FE for metro status, state FE, survey year FE. Mean is reported for children in families where neither parent has an SCDR.

S5 Table. Heterogeneity by Race and Sex of Child (Veteran Sample).
