# Supplementary material for: The relationship between parental disability and child outcomes: Evidence from veteran Families
Source: PLoS One. 2022 Nov 9;17(11):e0275468. doi: 10.1371/journal.pone.0275468 (PMC9645595; doi:10.1371/journal.pone.0275468)
Supplement: S7 Table — (PDF) [file pone.0275468.s008.pdf]

	Sample: Father is a veteran		Sample: Mother is a veteran	
	Dep. Var.: Mother Works (1)	Dep. Var.: Mother's Hrs Worked (2)	Dep. Var. Father Works (3)	Dep. Var. Father's Hrs Worked (4)
Parent SCDR				
10 to 20 Percent	-0.003	-7.58	-0.013**	-81.95***
	(0.004)	(8.15)	(0.006)	(20.25)
30 to 40 Percent	-0.008*	-25.60**	-0.025***	-150.15***
	(0.005)	(10.66)	(0.008)	(26.19)
50 to 60 Percent	-0.032***	-75.12***	-0.026**	-147.67***
	(0.006)	(12.68)	(0.010)	(31.64)
70 Percent or Higher	-0.079***	-172.10***	-0.075***	-292.29***
	(0.005)	(9.49)	(0.009)	(26.53)
Observations	408,117	408,117	54,915	54,915
Mean of dep. var.	0.750	1285	0.937	2101
p-value for test that SCDR 10-20 = SCDR 70+	0.000	0.000	0.000	0.000

*** p<0.01, ** p<0.05, * p<0.1 Standard errors clustered at the household level. Omitted group: Children in families where neither parent has a disability rating (SCDR=0). Sample: all children ages 5-18 living with a veteran father in columns 1 and 3 and with a veteran mother in columns 2 and 4. Controls: age FE, gender, dummy variables for single race categories (white, black, Hispanic), household size FE, FE for birth order, FE for number of siblings, FE for number of grandparents in household, mother's and father's age, education, and marital status FE as well as indicators for whether mothers and father served in 2001 and later (including indicators for missing parental information), FE for metro status, state FE, survey year FE. Mean is reported for children in families where neither parent has an SCDR.

S7 Table. Parental Disability and the Labor Supply of the Non-Disabled Parent (Veterans Sample)
