# Supplementary material for: The relationship between parental disability and child outcomes: Evidence from veteran Families
Source: PLoS One. 2022 Nov 9;17(11):e0275468. doi: 10.1371/journal.pone.0275468 (PMC9645595; doi:10.1371/journal.pone.0275468)
Supplement: S8 Table — (PDF) [file pone.0275468.s009.pdf]

	In Private School	Any Disability
	(1)	(2)
Parent SCDR		
10 to 20 Percent	-0.001	0.004***
	(0.002)	(0.001)
30 to 40 Percent	-0.011***	0.006***
	(0.003)	(0.002)
50 to 60 Percent	-0.017***	0.010***
	(0.003)	(0.002)
70 Percent or Higher	-0.014***	0.025***
	(0.002)	(0.002)
Observations	465,053	481,725
Mean of dep. var.	0.125	0.0543
p-value for test that SCDR 10-20 = SCDR 70+	0.000	0.000

*** p<0.01, ** p<0.05, * p<0.1 Standard errors clustered at the household level. Omitted group: Children in families where neither parent has a disability rating (SCDR=0). Sample: all children ages 5-18 living with a household member that is a veteran. SCDR bin indicators are for the highest SCDR rating of any adult (age 18+) in the household. Controls: age FE, gender, dummy variables for single race categories (white, black, Hispanic), household size FE, FE for birth order, FE for number of siblings, FE for number of grandparents in household, mother's and father's age, education, and marital status FE as well as indicators for whether mothers and father served in 2001 and later (including indicators for missing parental information), FE for metro status, state FE, survey year FE, and county fixed effects. Mean is reported for children in families where neither parent has an SCDR.

S8 Table. Effects of Parent SCDR Controlling for County Fixed Effects (Veteran Sample).
